# Can self‐monitoring mobile health apps reduce sedentary behavior? A randomized controlled trial

**DOI:** 10.1002/1348-9585.12159

**Published:** 2020-08-26

**Authors:** Kojiro Yamamoto, Takeshi Ebara, Fumiko Matsuda, Tsuyoshi Matsukawa, Nao Yamamoto, Kenji Ishii, Takahiro Kurihara, Shota Yamada, Taro Matsuki, Naomichi Tani, Michihiro Kamijima

**Affiliations:** ^1^ Nagoya City University Graduate School of Medical Sciences Nagoya Japan; ^2^ The Ohara Memorial Institute for Science of Labour Tokyo Japan; ^3^ Faculty of Information Science Aichi Institute of Technology Toyota Japan; ^4^ Nagoya City University Graduate School of Economics Nagoya Japan; ^5^ The Association for Preventive Medicine of Japan Fukuoka Japan

**Keywords:** mobile health technology, sedentary behavior, self‐monitoring intervention

## Abstract

**Objective:**

To examine whether the self‐monitoring interventions of a mobile health app reduce sedentary behavior in the short and long terms.

**Method:**

We designed a double‐blind randomized control trial. Participants were selected from among the staff of a medical institution and registrants of an online research firm. Forty‐nine participants were randomly assigned to either a control group (n = 25) or an intervention group (n = 24). The control group was given only the latest information about sedentary behavior, and the intervention was provided real‐time feedback for self‐monitoring in addition to the information. These interventions provided for 5 weeks (to measure the short‐term effect) and 13 weeks (to measure the long‐term effect) via the smartphone app. Measurements were as follows: subjective total sedentary time (SST), objective total sedentary time (OST), mean sedentary bout duration (MSB), and the number of sedentary breaks (SB). Only SST was measured by self‐report based on the standardized International Physical Activity Questionnaire and others were measured with the smartphone.

**Results:**

No significant results were observed in the short term. In the long term, while no significant results were also observed in objective sedentary behavior (OST, MSB, SB), the significant differences were observed in subjective sedentary behavior (SST, β_int_ − β_ctrl_ between baseline and 9/13 weeks; 1.73 and 1.50 h/d, respectively).

**Conclusions:**

Real‐time feedback for self‐monitoring with smartphone did not significantly affect objective sedentary behavior. However, providing only information about sedentary behavior to users with smartphones may make misperception on the amount of their subjective sedentary behavior.

## 1 INTRODUCTION

In recent years, sedentary behavior, defined as sitting or reclining, and expending less than 1.5 metabolic equivalents (METs) of energy, has attracted interest as an independent predictor of cardio‐metabolic risk factors, type 2 diabetes, and all‐cause mortality.^1^, ^2^, ^3^ A systematic review showed that sitting for 9.5 hours or more per day increased the hazard ratio of mortality.^1^ Previous studies report that the mean sitting time per day of American office‐workers was 10.6 hours and that the mean sitting time per day of Japanese office‐workers was 6.4 hours during work time and 4.8 hours during leisure time.^4^, ^5^ Thus, disseminating knowledge about and promoting public understanding of the risk a sedentary lifestyle poses for human health has become an urgent challenge worldwide.

Sedentary behavior is measured in two major ways: (a) subjective measured time, a measure that is self‐reported by respondents and (b) objective measured time, a measure that uses the accelerometers of dedicated devices or built‐in smartphone sensors. Self‐reports of sedentary activity have been found to underestimate the actual sedentary time.^6^, ^7^, ^8^ Meanwhile, objective measures of sedentary time using an accelerometer of a dedicated device can also induce some biases; subjects inexperienced with wearing a device on their body or subjects wearing an unusual device may be more aware of its measurement and may thus engage in better behavior than that typical to their daily life. It is important to note here that the smartphones used in everyday life around the world can track users' daily activity (even if users are not aware of it) using multiple built‐in sensors, such as an accelerometer, a gyro sensor, and a GPS, and can also collect self‐reported data using questionnaires.^9^, ^10^, ^11^, ^12^ However, the objective measurements taken by smartphones also have particular biases that must be solved. Because the technology assumes that subjects place their phones in a chest pocket, for example, placing the phone on a charger or in a bag may cause it to overestimate sedentary time.^11^With subjective and objective measurements demonstrating many merits and demerits, it is better to perform composite measurements when possible.

Recent studies on effective interventions for reducing sedentary time focus on encouraging behavioral changes using information, feedback, and self‐monitoring.^13^, ^14^ In recent years, wearable devices and smartphones have gained attention as mobile health technologies that can promote behavioral changes.^15^, ^16^, ^17^ For example, smartphones promise to help users reduce sedentary time by monitoring their activity levels and providing real‐time, on‐screen feedback of current activity level. A previous intervention study with 58 Belgian workers showed that receiving information and feedback about their sedentary patterns helped users reduce their sedentary behavior.^15^ However, due to the high heterogeneity within “intervention,” such as type of intervention, intervention term (eg, short term or long term), and differences between intervention settings (eg, positive control or reference without intervention), evidence remains limited and difficult to generalize.^17^ Responding to this gap in the scholarly archive, this study aims to examine the short‐ and long‐term effects of self‐monitoring mobile health app interventions on subjective‐ and objective‐measured sedentary behaviors.

## 2 METHODS

This study was approved by the Institutional Review Board of Nagoya City University Graduate School of Medical Sciences (No. 46‐19‐0002).

### 2.1 Study sample

The participants were recruited from the staff of a medical checkup institution and persons registered as monitors with an online research firm. The former group was allocated to a short and long term (5‐ and 13‐week intervention, respectively) and the latter only a short‐term including 1 week of baseline assessments. Individuals were eligible to participate in the study if they were at least 20 years of age, had a desk‐bound job, worked at least 40 hours per week, were permitted to carry a smartphone and leave their seats freely during worktime, and were users of Android smartphones. Those who attended a hospital and pregnant woman were excluded. As for the recruitment from the online research firm, a total of 14 602 of the 15 258 people registered were excluded because they did not meet our eligibility requirements (n = 6310) or did not respond to our invitation letter (n = 8292). Ultimately, 656 applicants responded. Of these, 30 applicants were chosen at random.

**FIGURE 1:**
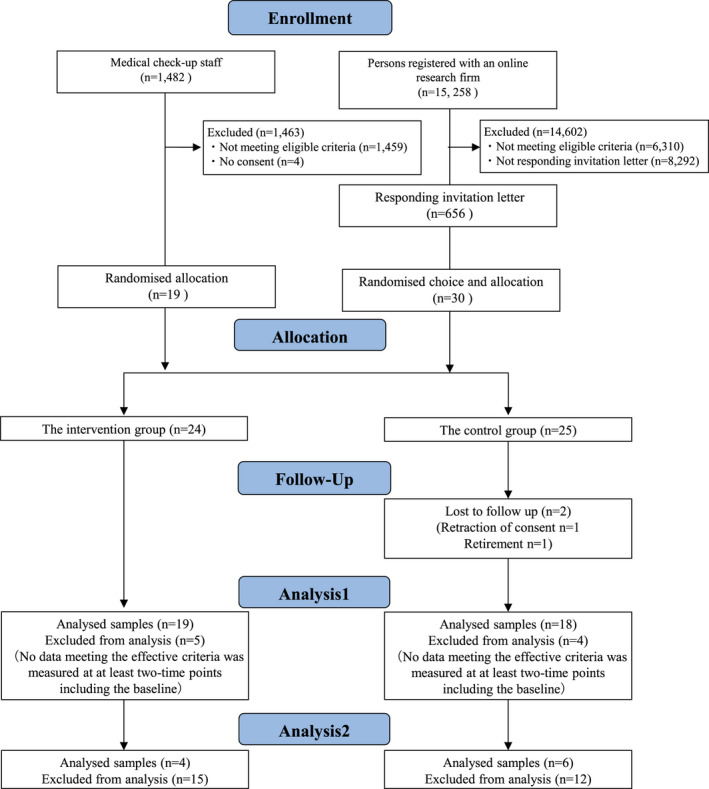
Participant selection flowchart

Participants from the medical checkup institution were recruited by a research collaborator. A total of 33 of 1482 employees who matched our criteria were provided with the details of the study. Of these, 19 people gave consent. Consequently, 49 participants in total were randomly assigned to a control group (n = 25) or an intervention (n = 24) (Figure 1).

### 2.2 Mobile health app specification

Our in‐house development mobile health app, Motion Logger ver. 1.5, measures time‐series log data obtained from embedded hardware sensors in smartphones, such as a step‐counting sensor and gyro sensor. The information from the built‐in sensors can be simultaneously measured at a sampling rate of 10 seconds during targeted measurement weeks (mentioned later) and transferred to a cloud server. The reliability of the data obtained from the app was confirmed by the test of the false detection rate during various sitting situations.^11^ The app also provides useful tips on sedentary‐related issues, a questionnaire for self‐reported sedentary time, and real‐time feedback of users' current activity levels.

### 2.3 Procedures

The short‐ and long‐term effects of a self‐monitoring mobile health app intervention on reducing sedentary behavior were evaluated in a randomized control trial design. After giving written informed consent, each eligible participant was asked to install the app in their smartphone and provide their demographic information prior to the baseline measurement.

**FIGURE 2:**
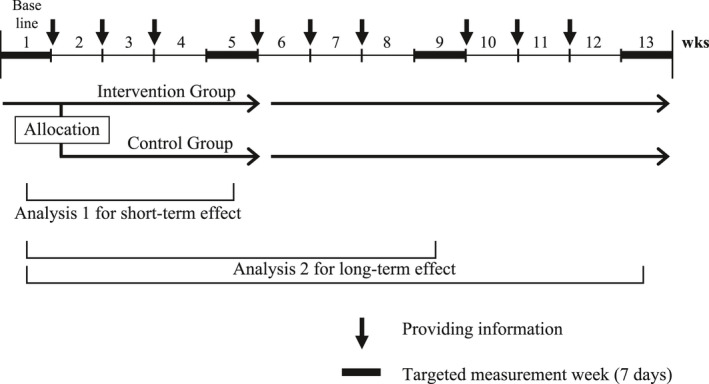
Scheme of intervention protocol for the RCT

The targeted measurement weeks set the short‐term effect at 5 weeks (Analysis 1) and the long‐term effect at 9 and 13 weeks (Analysis 2), following 1 week of baseline assessment (Figure 2). After the week of baseline measurement, the participants from each cluster of recruitment were randomly allocated into control or intervention groups with an allocation ratio of 1:1. Group allocations were double‐blinded (ie, concealed from participants and researchers). They were requested to wear their own smartphone in their pocket (chest pocket or pants pocket) or with neck strap during targeted measurement weeks except while in bed for objective measurements.

### 2.4 Intervention

#### 2.4.1 The latest information on sedentary time

This information was provided every week via the app. The information contains knowledge on the adverse effects of sedentary behavior, the advantages of regular interruptions of prolonged sitting, and tips for reducing sedentary time. The participants could read it at any time.

#### 2.4.2 Real‐time feedback for self‐monitoring

Push notifications provided daily real‐time feedback of users' current activity levels such as sedentary time and steps. The participants could confirm the graphical and analytical data of their current and recorded daily activity levels on the app at any time as part of their self‐monitoring.

The control group was given only the former set of information as positive control while the intervention group was given both. Both the control and intervention groups were asked to check the app every day, and the number of checking days divided by the period between measurements (21 days) was defined as the engagement rate (adherence rate).

### 2.5 Sample size estimation

Sample sizes were estimated based on mean differences and standard deviations shown in the previous study, under the condition of an unpaired *t* test with Bonferroni's correction.^15^ Based on the effect size of the total sedentary time (n = 66 and 31, mean values = 658.1 and 593.4, SD = 66.0 and 111.5) = 0.7 (Cohen's d), α = 0.016, we estimated that over 44 subjects were required to achieve at least 80% statistical power.

### 2.6 Data processing for analysis

In this study, a period of at least 1 minute of 0 steps was defined as sedentary behavior. Hours of sleep or missing data were excluded according to the participants' self‐reports. In addition, a period of at least 150 consecutive minutes of data of less than 1 m/s^2^ acquired from linear‐acceleration sensors was regarded as non‐measurement time and excluded.^18^, ^19^

The effective criteria day of sensor data were empirically at least 8 h/d, more than 60% of the total amount of data, more than 1000 steps, and workday.^20^ Participant data meeting the effective criteria were analyzed at least two‐time points including the baseline.

Since subjective total sedentary time (SST), objective total sedentary time (OST), and sedentary breaks (SB) were affected by measurement time, these indicators were adjusted to mean measurement time using the residuals obtained from linear regression models.^21^, ^22^

### 2.7 Sedentary behavior outcomes

The following indicators were adopted as primary outcomes; reliability was confirmed for all indicators with the exception of SST. For details about the definition and calculation of these outcomes, refer to the previous study.^20^

#### 2.7.1 SST: Subjective total sedentary time (hours/day)

The total sitting time obtained from participants' self‐report based on the standardized International Physical Activity Questionnaire (IPAQ; “how much time did you spend sitting yesterday?”) via the app, which was responded every morning during the measurement weeks.^23^

#### 2.7.2 Objective total sedentary time (h/d)

The total time of sedentary bout duration over 10 minutes.

#### 2.7.3 Mean sedentary bout duration (min/d)

The mean of sedentary bout duration over 10 minutes.

#### 2.7.4 Sedentary breaks (number of times/d)

The number of transitions from 0 to 1 step or more.

### 2.8 Statistical analysis

A linear mixed model analysis (LMM) was used to examine the intervention effect of the self‐monitoring intervention on sedentary behavior.^15^, ^24^, ^25^ The analysis included the condition of intervention (control, intervention) and measurement points (baseline, 5 weeks, 9 weeks, and 13 weeks). Both intervention and measurement points were treated as fixed effects in the model, and individuals were treated as a random effect. We calculated each partial regression coefficient of the control group (β_ctrl_) and the intervention group (β_int_). Partial regression coefficients indicate slopes yielding from differences of pairs between the baseline and each measurement point. If the differences of coefficients (β_int_ − β_ctrl_) are not equal to 0, then the result indicates a statistical intervention effect. This also shows the interaction of each measurement point with the intervention. To confirm the differences between intervention terms between the 5‐ and 13‐week groups affecting on psychobehavioral response, a sensitivity subanalysis was conducted between recruitments at the 5‐week point. Significance was set at *P* < .05. Data processing and analyses were conducted using R Version 3.5.0.

## 3 RESULTS

### 3.1 Participant characteristics

The number of participants for Analysis 1 for the short‐term effect was 37 (control group: n = 19, intervention group: n = 18, Table 1). Participants had a mean age of 43.2 ± 8.8 years, a mean BMI of 22.4 ± 3.3 kg/m^2^, and a mean measurement time of 15.4 ± 3.0. The mean of indicators of sedentary behavior at baseline were 9.4 ± 2.2 h/d for SST, 10.8 ± 1.9 h/d for OST, 41.7 ± 14.7 min/d for MSB, and 36.1 ± 14.7 times/d for SB. The control group had a longer measurement time and OST than the intervention group.

**TABLE 1:** Characteristics of participants

	Control group	Intervention group	Total	*P*
All samples for analysis 1 (short‐term effect)	n = 19	n = 18	n = 37	
Demographic characteristics				
Age (y)^a^	43.1 (9.9)	43.3 (7.7)	43.2 (8.8)	.92
Sex: male^b^	9 (47.4)	5 (27.8)	14 (37.8)	.31
BMI (kg/m^2^)^a^	22.5 (3.4)	22.2 (3.3)	22.4 (3.3)	.93
METs (/wk)^a^	394.4 (555.9)	140.6 (202.8)	267.5 (431.7)	.27
Measuring time (h/d)^a^	14.6 (3.1)	16.0 (2.8)	15.4 (3.0)	.01*
Indicators of sedentary behavior^a^				
SST (h/d)	9.6 (2.4)	9.3 (2.0)	9.4 (2.2)	.44
OST (h/d)	10.4 (1.8)	11.1 (1.9)	10.8 (1.9)	.03*
MSB (min/d)	40.4 (12.7)	42.8 (16.3)	41.7 (14.7)	.34
SB (number of times/d)	38.2 (12.6)	36.1 (14.7)	37.1 (13.7)	.40
Only participants from medical checkup institution staff group for Analysis 2 (long‐term effect)	n = 6	n = 4	n = 10	
Demographic characteristics				
Age (y)^a^	43.7 (9.8)	46.2 (11.0)	44.7 (9.8)	.77
Sex: male^b^	3 (50.0)	2 (50.0)	5 (50.0)	1.00
BMI (kg/m^2^)^a^	21.7 (2.4)	22.1 (5.8)	21.9 (3.8)	.70
Mets per weeks (mets/wk)^a^	45.0 (90.0)	20.0 (21.2)	32.5 (62.0)	.49
Measuring time (h/d)^a^	12.7 (3.2)	15.0 (3.4)	13.9 (3.4)	.01^*^
Indicators of sedentary behavior^a^				
SST (h/d)	11.1 (2.4)	9.4 (1.6)	10.5 (2.3)	.44
OST (h/d)	11.5 (0.9)	10.3 (1.5)	10.9 (1.4)	<.01*
MSB (min/d)	42.8 (10.8)	32.8 (9.4)	37.4 (11.2)	.01^*^
SB (number of times/d)	32.3 (8.1)	44.4 (9.8)	38.7 (10.8)	.00^*^

Abbreviations: BMI, body mass index; METs, metabolic equivalents; MSB, mean sedentary bout duration; OST, objective total sedentary time; SB, sedentary breaks; SST, subjective total sedentary time.

^a^ Mean (SD).

^b^ n (%).

* *P* < .05.

Meanwhile, the number of participants for Analysis 2 for the long‐term effect was 10 (control group: n = 6, intervention group: n = 4). Participants had a mean age of 44.7 ± 9.8 years, a mean BMI of 21.9 ± 3.8 kg/m^2^, and a mean measurement time of 13.9 ± 3.4. The mean of indicators of sedentary behavior at the baseline were 10.5 ± 2.3 h/d for SST, 10.9 ± 1.4 h/d for OST, 37.4 ± 11.2 min/d for MSB, and 38.7 ± 10.8 times/d for SB. The intervention group had a longer measurement time, OST, and MSB and smaller SB than the control group.

### 3.2 Engagement rate

Table 2 shows the engagement rate of each group by measurement point. According to the results of a two‐way analysis of variance (factors included the measurement points and condition of intervention), the intervention group had a higher engagement rate than the control group (*F* = 5.3, *P* = .03). Notably, both engagement rates tended to decrease by measurement point.

**TABLE 2:** Engagement rate: % (SD) in control and intervention groups

Measurement points	Condition of intervention	
	Control group	Intervention group
2‐4 wks	67.7 (34.9)	88.5 (22.6)
6‐8 wks	50.5 (45.4)	66.7 (45.9)
10‐12 wks	44.8 (40.5)	60.3 (43.2)

### 3.3 Short‐term and long‐term effects of intervention

Table 3 shows the result of LMM. Actual sample sizes for each analysis given as person‐time are also shown in Table 3. All the differences of coefficients (β_int_ − β_ctrl_) at 5 weeks have a 95% confidence interval including 0, indicating that the short‐term effects (between the baseline and 5 weeks) of self‐monitoring intervention were not observed.

**TABLE 3:** Short‐term (between baseline and 5 weeks) and long‐term (between baseline and 9/13 weeks) effects of self‐monitoring intervention on reducing sedentary behavior

	Estimated coefficient (β) (95% CI)						Difference of coefficients (β_int_ − β_ctrl_)		
	Control group (β_ctrl_)			Intervention group (β_int_)					
	5 wks	9 wks	13 wks	5 wks	9 wks	13 wks	5 wks	9 wks	13 wks
All samples									
Sample size	n = (92, 56)	n = (13, 3)	n = (22, 10)	n = (90, 56)	n = (16, 7)	n = (19, 11)			
SST (h/d)	−0.45 (−0.99 to 0.09)	−0.68 (−1.83 to 0.47)	−**1.15** **(−2.10 to** −**0.21)**	0.14 (−0.41 to 0.69)	1.06 (−0.04 to 2.16)	0.36 (−0.68 to 1.39)	0.59 (−0.18 to 1.37)	**1.73** **(0.14‐3.32)**	**1.50** **(0.10‐2.91)**
OST (h/d)	−0.03 (−0.5 to 0.45)	0.01 (−1.56 to 1.58)	−0.09 (−1.06 to 0.88)	−0.03 (−0.49 to 0.43)	1.23 (0.15‐2.31)	0.81 (−0.10 to 1.72)	0.00 (−0.66 to 0.67)	1.24 (−0.67 to 3.15)	0.91 (−0.42 to 2.25)
MSB (min/d)	0.85 (−3.11 to 4.81)	1.94 (−11.09 to 14.96)	4.16 (−3.83 to 12.15)	2.02 (−2.37 to 6.42)	16.76 (6.51‐27.0)	8.26 (−0.34 to 16.86)	1.11 (−4.84 to 7.06)	14.59 (−2.49 to 31.67)	4.07 (−7.83 to 15.97)
SB (number of times/d)	−0.22 (−3.56 to 3.12)	−4.23 (−15.3 to 6.83)	−5.45 (−12.31 to 1.41)	−2.00 (−5.54 to 1.53)	−12.23 (−20.48 to −3.98)	−9.56 (−16.5 to −2.63)	−1.76 (−6.64 to 3.12)	−8.00 (−22.05 to 6.05)	−4.17 (−14.00 to 5.65)
Only participants from online research firm									
Sample size	n = (67, 46)			n = (72, 48)					
SST (h/d)	−0.07 (−0.66 to 0.51)	—	—	−0.01 (−0.66 to 0.64)	—	—	0.06 (−0.81 to 0.93)	—	—
OST (h/d)	0.14 (−0.44 to 0.71)	—	—	−0.09 (−0.69 to 0.51)	—	—	−0.22 (−1.05 to 0.62)	—	—
MSB (min/d)	0.46 (−4.05 to 4.98)	—	—	1.36 (−4.24 to 6.96)	—	—	0.80 (−6.32 to 7.93)	—	—
SB (number of times/d)	0.02 (−3.99 to 4.03)	—	—	−1.4 (−6.14 to 3.34)	—	—	−1.38 (−7.55 to 4.79)	—	—
Only participants from medical checkup institution staff							—		
Sample size	n = (25, 10)			n = (18, 8)					
SST (h/d)	−1.23 (−2.32 to −0.14)	—	—	0.41 (−0.65 to 1.47)	—	—	**1.64** **(0.11‐3.17)**	—	—
OST (h/d)	−0.61 (−1.32 to 0.11)	—	—	0.11 (−0.63 to 0.85)	—	—	0.74 (−0.41 to 1.89)	—	—
MSB (min/d)	3.54 (−4.59 to 11.67)	—	—	3.17 (−3.28 to 9.62)	—	—	−0.12 (−10.88 to 10.63)	—	—
SB (number of times/d)	−0.89 (−6.99 to 5.22)	—	—	−3.04 (−8.22 to 2.13)	—	—	−2.12 (−10.56 to 6.31)	—	—

Values in bold shows *P* < .05, Sample size: n = person‐time (subjective measurement, objective measurement).

Abbreviations: 95% CI, 95% confidence interval; MSB, mean sedentary bout duration; OST, objective total sedentary time; SB, sedentary breaks; SST, subjective total sedentary time.

Likewise, as for the long‐term effect between the baseline and 9/13 weeks, significant intervention effects were not observed with the exception of differences of β_int_ − β_ctrl_ (baseline and 9/13 weeks) in SST (1.73 and 1.50 h/d, respectively). Coefficient β, which indicates the slopes of the regression lines of SST under each group, was largely negative (below 0) in the control group at 5, 9, and 13 weeks (at −0.45, −0.68, and −1.15, respectively) and positive in the intervention group (at 0.14, 1.06, and 0.36).

### 3.4 Sensitivity analysis

To confirm differences between intervention terms, a subanalysis between the recruitments was conducted at the 5‐week point of measurement. The results of the analysis of the participants from the online research firm did not significantly differ from those of all samples. In contrast, in the participants from the medical checkup institution, intervention effects (β_int_ − β_ctrl_) were observed in SST at 5 weeks; this differs from the results of the analysis of all samples (Table 3). Table S1indicates the demographic characteristics of the two recruiting groups. No significant differences were found in demographics between groups except measuring time. We also confirmed the step counts as a physical activity level during the intervention period. No differences were found between the control and the intervention groups during the short‐ and long‐term periods.

## 4 DISCUSSION

This study was among the first to evaluate the impact of a self‐monitoring mobile health app intervention on reducing sedentary behavior. Our results showed that such a self‐monitoring intervention did not significantly affect objective sedentary behavior. A previous systematic review that combines the results of 16 RCT studies reported that self‐monitoring interventions reduced sedentary time by 34.4 min/d.^13^ Additionally, the recent Cochrane database of systematic reviews also showed that multicomponent interventions composed of coaching, self‐monitoring, education, and email messages reduced sedentary behavior (101 minutes/8 hours of work time).^14^ One reason for no significant effect of our intervention may be explained by the setting of the control group as a positive control. In this study, because both the control and the intervention groups were provided educational information for general sedentary behavior, all participants could not recognize their allocation. As such positive control provided, the self‐monitoring intervention succeeded in blinding. A previous study that set a positive control like ours, which was examining the impact of education and real‐time feedback with activity trackers compared to one without, similarly found no differences in their impacts on sedentary behavior.^25^ Another possible explanation could be found in that the combination of self‐monitoring and no‐personalized information of general sedentary time has no enough effect on reducing sedentary behavior or increasing physical activity (Table S2). For instance, a previous study indicated that the combination of self‐monitoring and personalized suggestions was increasing walking time compared with non‐personalized suggestions.^26^ Our results contribute to this knowledge, revealing that self‐monitoring intervention with a smartphone did not significantly reduce objective sedentary behavior.

Next, it is noteworthy that SST decreased at 9 and 13 weeks in our control group. A previous study revealed that office‐workers with a multicomponent intervention reduced their subjective sedentary time at 12 weeks from the baseline, while sedentary time measured objectively did not change^27^—this finding is consistent with our result. Besides, our results of the intervention group also indicated that self‐monitoring intervention with real‐time feedback could maintain the subjective estimation of sedentary time, not to making it underestimate like the control group. Some studies show that self‐reported sedentary time might be underestimated compared with objective measurement sedentary time.^6^, ^7^, ^8^ Thus, it should be noted that self‐reported sedentary time might not be reflected in objective measurements of sedentary time and that sedentary behavior could thus be underestimated by intervention without self‐monitoring or feedback on sedentary time. The effect of intervention might not be the same between subjective sedentary behavior and objective sedentary behavior, and using both measurements may play a significant role in inquiries into the research findings on sedentary behavior.

### 4.1 Strengths and limitations

The first strength of this study was that it was the first study to examine the impact of the intervention on reducing sedentary behavior in workplaces with a randomized control trial in Japan. Because there is a limited number of studies regarding sedentary behavior, the present study significantly contributes to the occupational health field. Besides, our use of a double‐blinded randomized control trial was also strength in light of reducing biases. One other strength was our use of a smartphone to conduct an objective measurement of sedentary behavior. In a previous systematic review regarding mobile health interventions, a clear decline in the engagement of long‐term intervention and measuring with dedicated devices was reported.^17^ Because the smartphone was used in every life, it may have an advantage for a long‐duration study. Additionally, we used recently validated indicators of sedentary behavior, such as the total time of sedentary bout duration over 10 minutes. A previous study indicated that a short sedentary bout duration might be regarded as a physical activity indicating frequent changes between sitting and standing, rather than a state‐like sedentary behavior.^20^ Therefore, it may be valid to use indicators calculated by excluding bout durations under 10 minutes.

This study also had some limitations. First, there might be some kind of effect on the result due to the small number of participants. Differences were observed between the control group and the intervention group in some variables at the baseline. In particular, the participants of Analysis 2 of the long‐term effect may have been potentially biased due to their small number. Simple randomization is more appropriate in trials with larger samples; in future studies, we will employ a more appropriate randomization method. Second, this study examined the effect of the intervention over only very specific short‐term (5 weeks) and long‐term (13 weeks) periods. Thirteen weeks might have been an insufficient period for evaluating the long‐term intervention effect. Also, “acceptability,” which may be an important factor in examining the effects of long‐term interventions on behavior,^28^ was not measured. Third, the data could be measured while participants wore their phones. The no‐measurable situation, especially during leisure, might be occurred, and these data were excluded as non‐measurement time. This might become common problems in the study using the data obtained from the smartphone,^11^, ^20^ whereas many previous studies used specific devices. Although some studies examined the availability of smartphone measurements for academic research, the data compatibility with respect to the measurement of SB is not guaranteed between a smartphone and the specific wearable devices.^11^, ^12^

Finally, our participants were all adult office‐workers. The intervention could be varied by selecting more heterogenous participants by changing, for example, selection criteria related to age and working environment. Therefore, any generalizations of our findings should be made with caution.

## 5 CONCLUSION

We conclude that providing real‐time feedback for self‐monitoring activity levels with a smartphone did not significantly affect users' objective sedentary behavior. In contrast, providing only information about sedentary behavior to users may make misperception on the amount of their subjective sedentary behavior at 9 and 13 weeks from the baseline. Thus, further research that carefully considers informational bias and uses composite measurements is needed.

## DISCLOSURES

Approval of the research protocol: This study was approved by the Institutional Review Board of Nagoya City University Graduate School of Medical Sciences (No. 46‐19‐0002). Informed consent: Written informed consent was obtained from all participants in this study. Conflict of interest: The authors have no conflict of interests to declare for this study.

## AUTHOR CONTRIBUTIONS

TE, FM, TM, and NY designed and developed the study protocol and the measurement method. KY analyzed the data and wrote the first draft of the manuscript. KI, TK, SY, and TM supported the analysis and interpreted the data. MK and TE supervised KY with respect to ergonomics and occupational health. TE was responsible for the study as PI and assisted with the drafting of the manuscript. All the authors interpreted the data, contributed to the writing of the manuscript, critically revised it for important intellectual content, and agreed with the final version and the findings.

## Supporting information

Tables S1‐S2
