# Supplementary material for: Can self‐monitoring mobile health apps reduce sedentary behavior? A randomized controlled trial
Source: J Occup Health. 2020 Aug 26;62(1):e12159. doi: 10.1002/1348-9585.12159 (PMC7448798; doi:10.1002/1348-9585.12159)
Supplement: Supplementary file 1 — Tables S1‐S2 [file JOH2-62-e12159-s001.docx]

Supplementary Table 1. Demographic characteristics of two recruiting groups

|  | Online research firm group | | Medical check-up institution staff group | | Total | | *p* |  |
| --- | --- | --- | --- | --- | --- | --- | --- | --- |
|  | n=27 | | n=10 | | n= 37 | |  |  |
| Age (years)^†^ | 42.2 | (8.9) | 45.0 | (8.5) | 43.2 | (8.8) | 0.37 |  |
| Sex: male ^‡^ | 8 | (33.3) | 6 | (46.2) | 14 | (37.8) | 0.50 |  |
| BMI (kg/m^2^) ^†^ | 22.3 | (3.2) | 22.5 | (3.6) | 22.4 | (3.3) | 0.57 |  |
| METs (/week)^†^ | 342.2 | (493.8) | 111.4 | (197.1) | 267.5 | (431.7) | 0.15 |  |
| Measuring time (hours/day) ^†^ | 15.8 | (2.8) | 14.4 | (3.4) | 15.4 | (3.0) | 0.02 | ^*^ |

* *p*<0.05, †Mean(SD), ‡n(%). SST, subjective total sedentary time; OST, objective total sedentary time; MSB, mean sedentary bout duration; SB, sedentary breaks; BMI, body mass index; METs, metabolic equivalents.

Supplementary Table 2. Short-term (between baseline and 5 weeks) and long-term (between baseline and 9 /13 weeks) effects of self-monitoring intervention on step counts

|  | Estimated coefficient (β) (95%CI) | | | | | | |  | Difference of coefficients (β_int_ - β_ctrl_) | | |
| --- | --- | --- | --- | --- | --- | --- | --- | --- | --- | --- | --- |
|  | Control Group (β_ctrl_) | | |  | Intervention Group (β_int_) | | |  |  |  |  |
|  | 5 weeks | 9 weeks | 13 weeks |  | 5 weeks | 9 weeks | 13 weeks |  | 5 weeks | 9 weeks | 13 weeks |
|  | n = 18 | n = 6 | n = 6 |  | n = 19 | n = 4 | n = 4 |  |  |  |  |
| β of Step counts  (hours/day) ^a^ | 190.7  (-905.1 - 1286.4) | -49.8  (-3712.8 - 3613.2) | 2034.6  (-247.3 - 4316.6) |  | 655.1  (-124.3 - 1434.4) | -643.2  (-2459 - 1172.6) | 903.4  (-620.8 - 2427.7) |  | 468.5  (-862.7 - 1799.7) | -588.8  (-4436.7 - 3259.2) | -1127.8  (-3820.3 - 1564.8) |

^a^ Steps were adjusted to mean measurement time using the residuals obtained from linear regression models.
